# Steered molecular dynamic simulations reveal Marfan syndrome mutations disrupt fibrillin-1 cbEGF domain mechanosensitive calcium binding

**DOI:** 10.1038/s41598-020-73969-2

**Published:** 2020-10-08

**Authors:** Stephen J. Haller, Adrian E. Roitberg, Andrew T. Dudley

**Affiliations:** 1 Holland Regenerative Medicine Program, Department of Genetics, Cell Biology and Anatomy, University of Nebraska Medical Center, 985965 NE Medical Center, 6064 DRCII, Omaha, NE 68198-5965 USA; 2 Department of Chemistry, University of Florida, Gainesville, FL USA

**Keywords:** Molecular biophysics, Molecular modelling, Connective tissue diseases, Genetics

## Abstract

Marfan syndrome (MFS) is a highly variable genetic connective tissue disorder caused by mutations in the calcium binding extracellular matrix glycoprotein fibrillin-1. Patients with the most severe form of MFS (neonatal MFS; nMFS) tend to have mutations that cluster in an internal region of fibrillin-1 called the neonatal region. This region is predominantly composed of eight calcium-binding epidermal growth factor-like (cbEGF) domains, each of which binds one calcium ion and is stabilized by three highly conserved disulfide bonds. Crucially, calcium plays a fundamental role in stabilizing cbEGF domains. Perturbed calcium binding caused by cbEGF domain mutations is thus thought to be a central driver of MFS pathophysiology. Using steered molecular dynamics (SMD) simulations, we demonstrate that cbEGF domain calcium binding decreases under mechanical stress (i.e. cbEGF domains are mechanosensitive). We further demonstrate the disulfide bonds in cbEGF domains uniquely orchestrate protein unfolding by showing that MFS disulfide bond mutations markedly disrupt normal mechanosensitive calcium binding dynamics. These results point to a potential mechanosensitive mechanism for fibrillin-1 in regulating extracellular transforming growth factor beta (TGFB) bioavailability and microfibril integrity. Such mechanosensitive “smart” features may represent novel mechanisms for mechanical hemostasis regulation in extracellular matrix that are pathologically activated in MFS.

## Introduction

Marfan syndrome (MFS) is an autosomal dominant connective tissue disorder that affects multiple body systems and organs^1^. The disorder is caused by mutations in *FBN1*, which encodes the calcium-binding extracellular matrix glycoprotein fibrillin-1^2,3^. Fibrillin-1 forms the principal component of 10–12 nm diameter microfibrils, which are essential for elastic fiber assembly and structure^4,5^. Connective tissue weakness caused by low-quality or decreased numbers of microfibrils is responsible for the primary clinical problems associated with MFS, including aortic aneurysm, dissection, and rupture^6,7^.

Although the natural history of MFS is weakened connective tissues, clinical presentations are highly variable. In contrast to classical MFS (cMFS), patients with the most severe form (neonatal MFS; nMFS) rarely survive past the second year of life^8,9^. Previous studies demonstrated nMFS mutations tend to localize in an internal region of *FBN1* called the neonatal region^10–12^. This region primarily encodes eight calcium-binding epidermal growth factor-like (cbEGF) domains, each of which binds one calcium ion and is stabilized by six highly conserved cysteine residues that form three disulfide bonds in a C1–C3, C2–C4, and C5–C6 arrangement^13^. Crucially, calcium plays a fundamental role in modulating the biophysical properties of fibrillin-1^14–16^. Bound calcium stabilizes cbEGF domains and cbEGF-cbEGF inter-domain interfaces, extending tandem cbEGF domain repeats into rigid rod-like structures^11^. Calcium protects cbEGF domains from proteolytic degradation and facilitates protein–protein interactions crucial for microfibril integrity^14,16,17^. cbEGF domain mutations causing MFS are thus presumed to interfere with calcium binding, thereby perturbing microfibril assembly, structure, and function^18^.

Despite identification of a neonatal region, genotype/phenotype associations in MFS are not absolute. Variable expressivity of identical mutations, including cbEGF domain mutations in the neonatal region, produce both cMFS and nMFS phenotypes^19^. Based on the biophysical importance and state-dependence of bound calcium, we hypothesized that changes in cbEGF domain conformation induced by mechanical stress couple with cbEGF domain mutations to variably influence calcium binding dynamics, which in turn governs MFS severity. This idea represents a leap forward from previous studies, which largely assessed cbEGF mutations at stress-free equilibrium. Direct assessment of non-equilibrium cbEGF domain calcium binding dynamics thus provides a novel framework for elucidating how mechanical stress (i.e. environment) in combination with mutations (i.e. genetics) influences genotype/phenotype variability in MFS.

Steered molecular dynamics (SMD) is a non-equilibrium enhanced sampling technique that applies time-varying energy potentials to computationally model protein unfolding over timescales accessible to molecular dynamics simulations^20^. In effect, SMD serves as a computational analogue for biophysical techniques such as atomic force microscopy (AFM) or optical tweezers. SMD is thus particularly well suited to investigate the molecular mechanisms through which mechanosensitive proteins respond to mechanical forces. Combined with the Jarzynski equality, non-equilibrium work profiles computed via SMD can be used to estimate free energy changes along unfolding pathways to construct equilibrium potential of mean force (PMF) profiles^21^. PMF profiles enable standard comparisons independent of unfolding rate, which converge on the reversible work profiles of the quasistatic unfolding process inaccessible to direct molecular dynamics simulation. SMD thus enables comparisons between cbEGF domain energy landscapes as these domains unfold under mechanical tension.

In this study, SMD simulations were performed to model the unfolding pathways and calcium binding dynamics of cbEGF domains under mechanical stress in the presence and absence of MFS mutations. We show that cbEGF domain calcium binding is mechanosensitive and that cbEGF disulfide bond mutations disrupt calcium-dependent unfolding. We additionally demonstrate that cbEGF domains are comparably stiff at low strain to regions presently believed to confer fibrillin-1 with most of its extensibility. We conclude by proposing a novel biophysical addition to the current MFS disease model that implicates the nano-mechanical environment of extracellular matrix as a crucial factor in microfibril function and an essential driver of MFS pathophysiology.

## Results

### Calcium predominantly increases low strain cbEGF domain stiffness

Given the essential role calcium plays in fibrillin-1 microfibrils, which serve as integral components of stretchable elastic fibers^4,5^, we were first interested in determining how wild-type cbEGF domains deform under mechanical stress with and without calcium. To achieve this, we first performed baseline molecular dynamics simulations at stress-free equilibrium. These simulations enabled practical validation of the molecular dynamics force field by comparing these results with previous biophysical findings measured at stress-free equilibrium. We then performed SMD simulations to model how cbEGF domains unfold under non-equilibrium conditions that mimic mechanical stretching. Although fibrillin-1 contains 43 cbEGF domains, the domain pair cbEGF12-cbEGF13 was chosen for primary investigation. A domain pair was chosen over a single domain because inter-domain effects are well documented. Further reasons for choosing cbEGF12-cbEGF13 included: (1) resides in the neonatal region, thus permitting subsequent investigation of nMFS and cMFS mutations; (2) cbEGF12 is the most frequently mutated domain in nMFS; and (3) solution NMR structures were publicly available (PDB ID: 1LMJ)^13^.

#### Stress-free equilibrium

**Figure 1:**
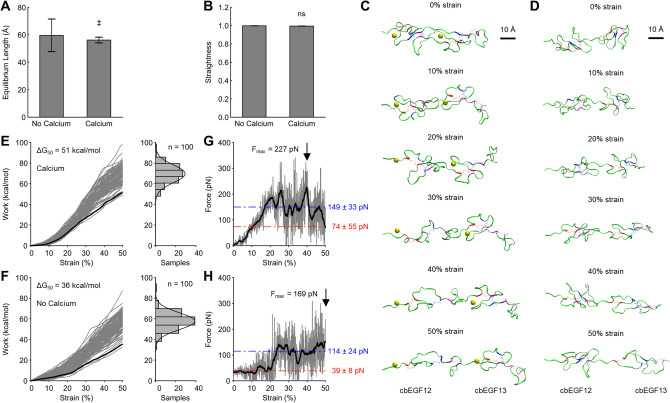
Calcium increases fibrillin-1 cbEGF domain stiffness predominantly at low strain. (**A**) Calcium increases cbEGF12-cbEGF13 length and decreases length variability at equilibrium. (**B**) Calcium has no effect on cbEGF12-cbEGF13 straightness. (**C**,**D**) Snapshots from cbEGF12-cbEGF13 SMD simulations captured between 0 and 50% strain with and without calcium, respectively (note: snapshots from lowest energy trajectory). (**E**,**F**) Left: work profiles (gray) and Jarzynski average (black) with and without calcium, respectively. Free energy change from 0–50% strain (ΔG_50_) indicated. Right: histograms of ΔG_50_ values with Gaussian distribution. (**G**,**H**) Force profile (gray) with smoothed profile (black) calculated using a moving average (10 ns width). Average force and standard deviation are shown over strain regions of 0–20% (red) and 20–50% (blue). n = 100 samples at a constant pull speed of 0.1 Å/ns with a spring constant of 7.4 kcal/mol/Å^2^. See Fig. S1 and S2 for validations. T-test used for mean comparisons. F-test used for standard deviation (SD) comparisons. ‡ p < 0.05 for mean and SD.

Following equilibration, the length and straightness of cbEGF12-cbEGF13 were sampled from an ensemble of isothermal-isobaric (NPT) trajectories (n = 10) with and without calcium. Removal of calcium led to a small yet significant decrease in length (59.60 ± 2.06 Å vs 56.19 ± 11.86 Å; p = 0.013) with a significant increase in standard deviation (F-test; p = 0.016) highlighting the stabilizing role of calcium (Fig. 1A). Neither the mean nor standard deviation for straightness (see “Methods”) was affected, indicating this variation in length was parallel to the long axis of these domains (Fig. 1B). Further analysis based on the methods by Adamovic et al.^22^ (see “Methods”) revealed calcium removal decreased cbEGF12-cbEGF13 equilibrium stiffness by ~ 60% (Table S1). Together, these results support previous findings that calcium maintains cbEGF domain repeats in extended rod-like conformations at stress-free equilibrium^11^.

Despite confirming most previous observations, the marginal decrease in length observed in these simulations (~ 6%) highlights an apparent paradox in the literature. Although removal of calcium has been measured to decrease cbEGF domain length by as much as 20–30%^23^, X-ray crystallography studies indicated calcium removal has minimal influence on local cbEGF domain structure^24^. We explain this discrepancy as an increase in cbEGF domain length variation caused by absence of calcium induced stability. We thus propose the 20–30% decrease in length measured without calcium using velocity sedimentation^23^ reflects the external effects of forces imparted by hydrodynamic drag that bias destabilized cbEGF domains to shorter lengths. Such an explanation underscores the important role mechanical forces play in combination with calcium in determining cbEGF domain structure and provides further rationale for exploring non-equilibrium dynamics induced by mechanical stress.

#### Non-equilibrium stretching

To determine how cbEGF domains respond to mechanical stress, we next performed SMD simulations to quantify the stiffness of cbEGF12-cbEGF13 with and without calcium (Fig. S1). Briefly, the N-terminal nitrogen was restrained in space (R = 100 kcal/mol/Å^2^) while a potential was applied between the N-terminal nitrogen and C-terminal carbon to stretch the domain pair to 150% of equilibrium length (i.e. 50% strain; ~ 60 Å to ~ 90 Å). This process was repeated (n = 100) at a constant rate of 0.1 Å/ns to gain adequate sampling to calculate a converged Jarzynski average corresponding to the potential of mean force (PMF) along the stretching reaction coordinate (Fig. S2). A maximum strain of 50% was chosen conservatively, as microfibrils are thought to stretch reversibly below strains of 50–80%^25,26^. While microfibril strain may not precisely correlate with cbEGF domain extension, this estimate provides a plausible upper limit of strain consistent with reversible deformation, which is fundamental to the physiologic deformation of elastic matrix. Snapshots of the calcium bound and calcium free structures stretched from 0–50% strain are shown (Fig. 1C,D; Video S1 and Video S2).

SMD simulations confirmed that bound calcium is a crucial modulator of the biophysical properties of cbEGF domains. Removal of calcium resulted in a downward shift of the non-equilibrium work profiles and decreased the change in free energy from 0–50% strain (∆G_50_) by 29% (51 kcal/mol vs 36 kcal/mol; Fig. 1E,F). Peak force was similarly reduced by 26% (227 pN vs 169 pN; Fig. 1G,H). Interestingly, whereas calcium bound cbEGF12-cbEGF13 exhibited a nearly linear increase in force from 0–20% strain, the calcium free form displayed an apparent stepwise change in force from 20–25% strain. Comparing force profiles between calcium bound and calcium free structures from 0–20% strain revealed a decrease in mean force of 47% (74 ± 55 pN vs 39 ± 8 pN; p < 0.001), whereas comparing from 20–50% strain revealed a decrease in mean force of 23% (149 ± 33 pN vs 114 ± 24 pN; p < 0.001) (Fig. 1G,H). Together, these results demonstrate the stiffening effects of calcium predominate at low strain and have less relative impact at high strain as cbEGF domains unfold. Indeed, stress-free equilibrium calculations appear to over-predict the stiffening role of calcium and are not sufficient alone to capture mechanically relevant changes in calcium induced stiffening that occur under non-equilibrium stretching.

### Strain decreases cbEGF domain calcium binding

**Figure 2:**
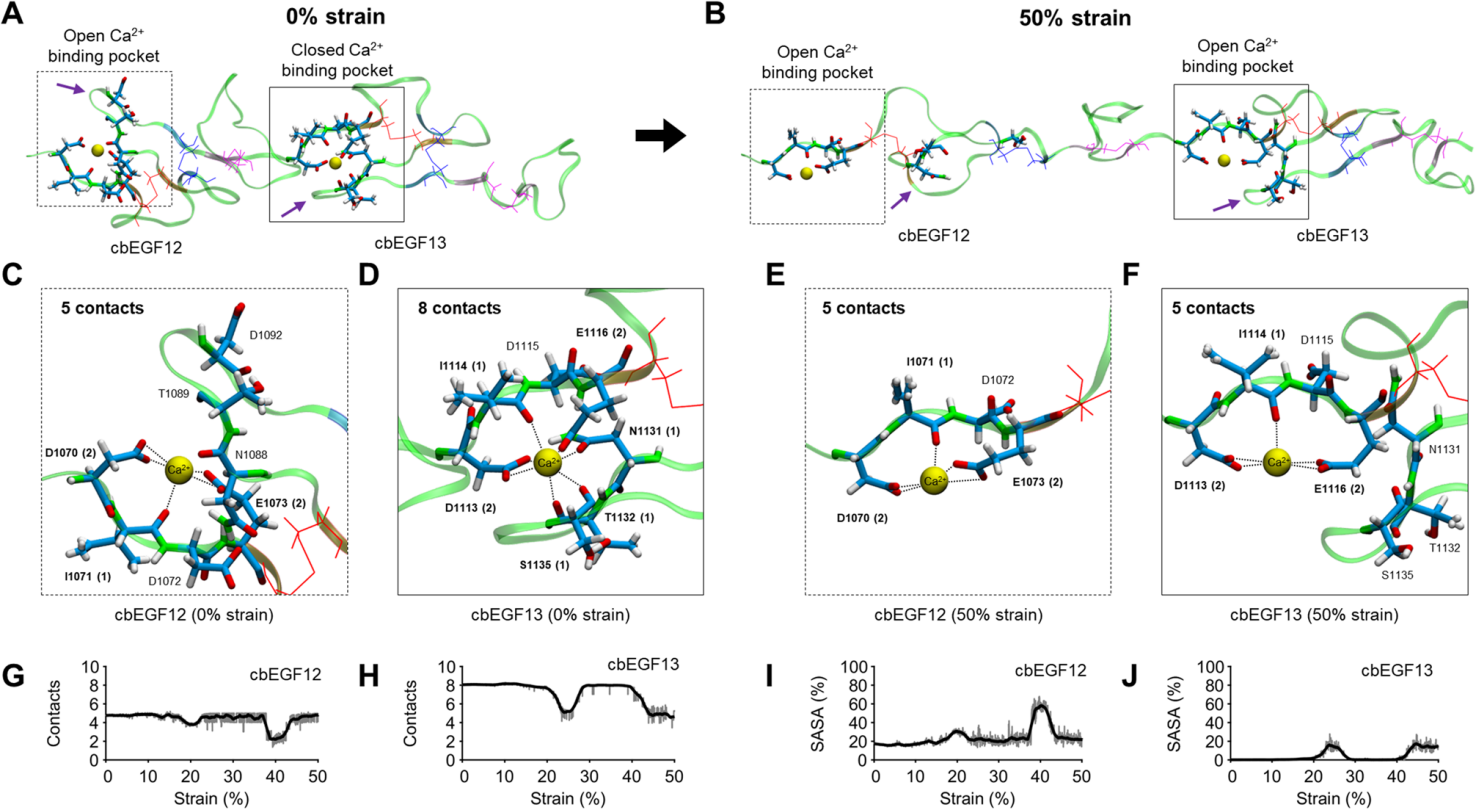
Fibrillin-1 cbEGF domain calcium binding is mechanosensitive. (**A**,**B**) Snapshots from lowest energy SMD trajectory reveal gross conformational changes in cbEGF12-cbEGF13 calcium binding pockets over 50% strain (~ 60 Å to ~ 90 Å). Purple arrows indicate β-hairpin of calcium binding pocket. (**C**,**D**) Calcium binding pockets are closed at 0% strain. Note: parenthesis indicate number of calcium-atom contacts < 4.0 Å. (**E**,**F**) Calcium binding pockets are open at 50% strain as calcium losses contact with β-hairpin (cbEGF12 residues N1088, T1089, and D1092; cbEGF13 residues N1131, T1132, and S1135) (**G**,**H**) Calcium-oxygen contacts decrease under strain. (**I**,**J**) Calcium solvent accessible surface area (SASA) increases under strain.

Given the predominant effect of calcium appears to be modulation of low strain cbEGF domain stiffness, we next wanted to characterize how cbEGF domain unfolding affects calcium binding. Qualitative analysis of cbEGF12-cbEGF13 stretched from 0–50% strain revealed large changes in calcium-binding pocket conformation (Fig. 2A,B). Closer inspection confirmed calcium maintains cbEGF domain conformation by coordinating key calcium binding residues as previously reported^13^. Interestingly, at stress-free equilibrium, cbEGF12 β-hairpin residues N1088, T1089, and D1092 were initially unbound from calcium; cbEGF12 thus had fewer initial calcium contacts than expected (Fig. 2C). Conversely, cbEGF13 was fully bound (Fig. 2D). These results were observed whether initial length restraints were included (i.e. for sampling consistent SMD snapshots; 4.80 ± 0.83 vs 8.04 ± 0.20; p < 0.001) or excluded (i.e. for establishing unbiased equilibrium lengths; 5.46 ± 0.42 vs 7.76 ± 0.164; p < 0.001). Together, these observations highlight previous findings that cbEGF-cbEGF inter-domain interfaces are crucial for stabilizing cbEGF domain calcium binding^27,28^.

Despite the observed differences in stress-free equilibrium calcium binding, stretching of cbEGF12-cbEGF13 produced open configurations in the calcium binding pockets for both cbEGF domains (Fig. 2E,F). For cbEGF12, unfolding caused marked displacement of β-hairpin binding residues (e.g. N1088, T1089, and D1092) and marked intra-domain unfolding (Fig. 2A–F). For cbEGF13, contacts between calcium and β-hairpin residues (e.g. N1131, T1131, S1135) were also lost, showing the destabilized features of cbEGF12 in the initial equilibrium condition (Fig. 2A–F). Quantification of calcium-oxygen contacts (Fig. 2G,H) and calcium solvent accessible surface area (SASA) (Fig. 2I,J) exponentially averaged over all trajectories confirmed these observations. Together, decreased calcium contacts concomitant with increased exposure of calcium to solvent suggest stress-induced cbEGF domain unfolding decreases calcium affinity. Importantly, these observations correlate with reduced protein stiffness as cbEGF12-cbEGF13 unfolded under stress.

### cbEGF12-cbEGF13 and TB4-cbEGF23 are similarly stiff at low strain

cbEGF12-cbEGF13 domain flexibility is unexpected given the claim of previous studies that the flexibility of fibrillin-1 in response to stretch results primarily from transforming growth factor beta (TGFB)-binding (TB)-cbEGF and hybrid-cbEGF domain interfaces^16,24,29,30^. Indeed, a fibrillin-1 molecule contains seven TB domains and two hybrid domains, each of which is followed by a cbEGF domain establishing a C-terminal interface (note: TB1 is an exception and is followed by the proline rich region). The claim of flexibility is thus rooted in these interfaces and is based on the flexible hydrophobic spring-like regions present between these domains and a calcium-dependent interface recoil mechanism^16^. Tandem cbEGF domain repeats were rejected as likely contributors to fibrillin-1 extensibility based on the observation that cbEGF repeats are ridged rod-like structures that are more extended when calcium bound^11,23^.

**Figure 3:**
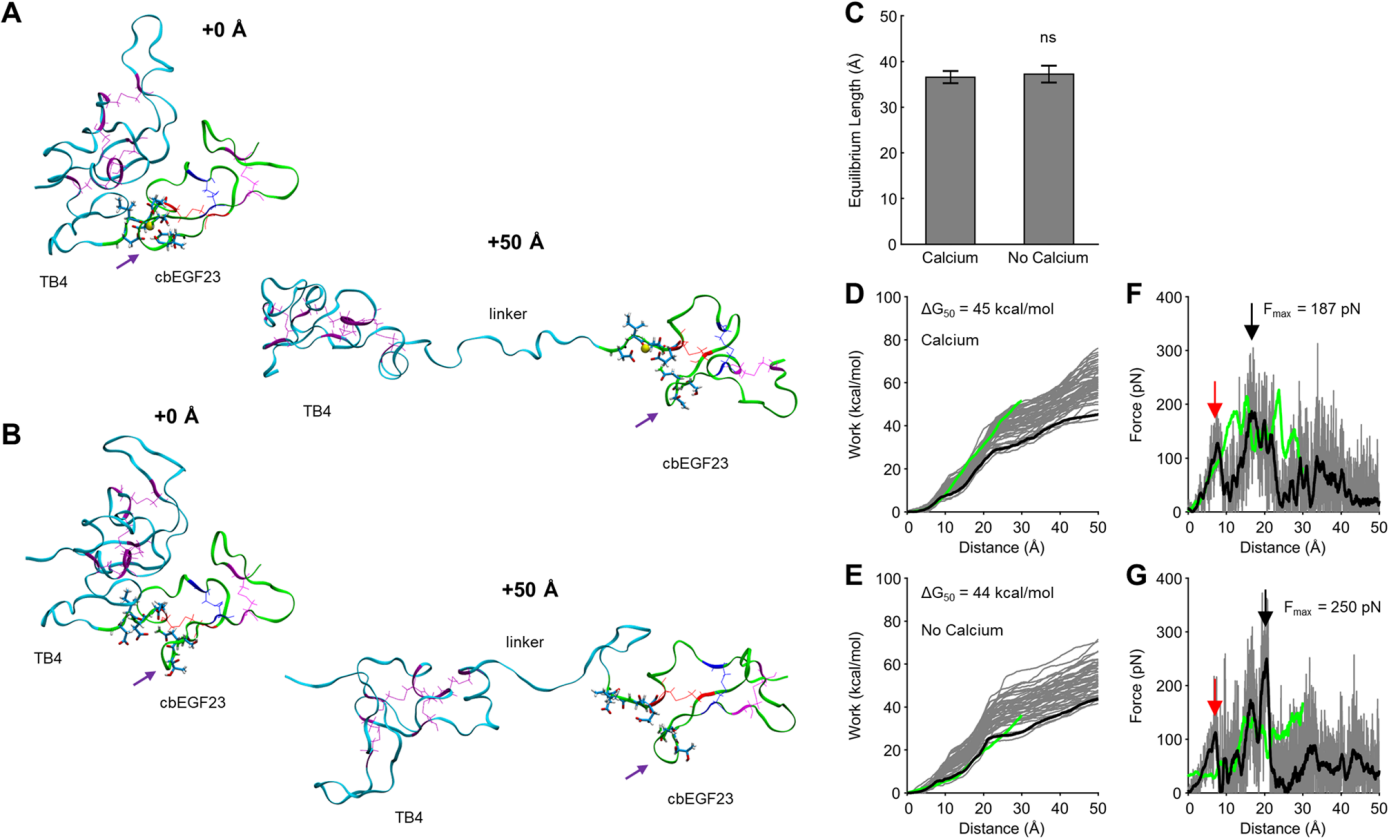
The interface between TB4 and cbEGF23 is calcium-independent and similarly stiff to cbEGF12-cbEGF13 at low strain. (**A**,**B**) Snapshots from TB4-cbEGF23 SMD simulations captured at + 0 Å and + 50 Å from mean equilibrium length. Purple arrow denotes cbEGF23 domain β-hairpin. (**C**) Calcium removal has no effect on TB4-cbEGF23 equilibrium length. (**D**,**E**) TB4-cbEGF23 work profiles (gray) and Jarzynski average (black) with and without calcium, respectively. cbEGF12-cbEGF13 profiles are overlaid in green. (**F**,**G**) TB4-cbEGF23 force profiles (gray) with smoothed profile (black) calculated using a moving average (10 ns width) with and without calcium, respectively. cbEGF12-cbEGF23 profiles are overlaid in green. Black and red arrows correspond to major and minor interface rupture peaks, respectively.

To assess the relative flexibility of cbEGF domain repeats, we performed additional SMD simulations on TB4-cbEGF23 (PDB ID: 1UZJ)^24^. Identical conditions as above were used for equal comparison, although a longer stretch distance of 50 Å was investigated, as previous studies have suggested TB-cbEGF interfaces can stretch up to 50 Å^24^. The resulting structural changes induced by stretching with and without calcium are shown (Fig. 3A,B; Video S3 and Video S4).

Surprisingly, calcium binding had no significant effect on TB4-cbEGF23 equilibrium length (36.58 ± 1.36 vs 37.24 ± 1.86; p = 0.285; Fig. 3C). Upon stretching, PMF profiles were markedly similar for TB4-cbEGF23 with and without calcium, with a decrease in the free energy change of only 2% (45 kcal/mol vs 44 kcal/mol; Fig. 3D,E). Here, each profile exhibited: (1) a minor peak at ~ 10 Å (red arrow) corresponding to partial rupture of the TB4-cbEGF23 inter-domain interface; (2) a major peak at ~ 20 Å (black arrow) corresponding to complete rupture of the TB4-cbEGF23 inter-domain interface; and (3) a plateau region following complete rupture corresponding to extension of the flexible spring-like linker region. Compared to cbEGF12-cbEGF13, which was only stretched to + 30 Å, the change in free energy of TB4-cbEGF23 was 35% lower with calcium (33 kcal/mol vs 51 kcal/mol; Fig. 3D) and 22% lower without calcium (28 kcal/mol vs 36 kcal/mol; Fig. 3E). Interestingly, peak rupture forces were also comparable between the calcium bound (187 pN vs 227 pN; Fig. 3F) and calcium free (250 pN vs 169 pN; Fig. 3G) forms. Collectively, these results demonstrate TB-cbEGF domain interfaces are similarly stiff to tandem cbEGF domains under low strain conditions. However, following inter-domain interface rupture, TB-cbEGF interfaces appear to have increased flexibility due to available slack from the predicted spring-like region. Conversely, tandem cbEGF domains appear to better maintain tension at high strain following calcium unbinding due to extensive disulfide bond crosslinks and lack of flexible linkers.

### TB4-cbEGF23 calcium binding decreases following inter-domain interface rupture

Despite distinct domain structures, we observed similar β-hairpin displacement and decreased calcium binding under stress in TB4-cbEGF23 as in cbEGF12-cbEGF13 (Fig. 3A). The conformational changes and calcium unbinding in both models thus supports a conserved mechanism for all fibrillin-1 cbEGF domains. Interestingly, β-hairpin displacement occurred under relatively low force (~ 20 pN) following extension of the flexible inter-domain linker in TB4-cbEGF23. This observation further supports the idea that inter-domain interfaces, including TB-cbEGF inter-domain interfaces, are crucial for stabilizing cbEGF domain calcium binding. Thus, cbEGF-cbEGF inter-domain interface stability and calcium affinity appear interrelated. However, given that stability of the TB4-cbEGF23 inter-domain interface was not appreciably affected by calcium, cbEGF domain calcium binding does not reciprocally stabilize inter-domain interfaces.

### nMFS and cMFS differ in cbEGF domain disulfide bond mutation frequency

**Figure 4:**
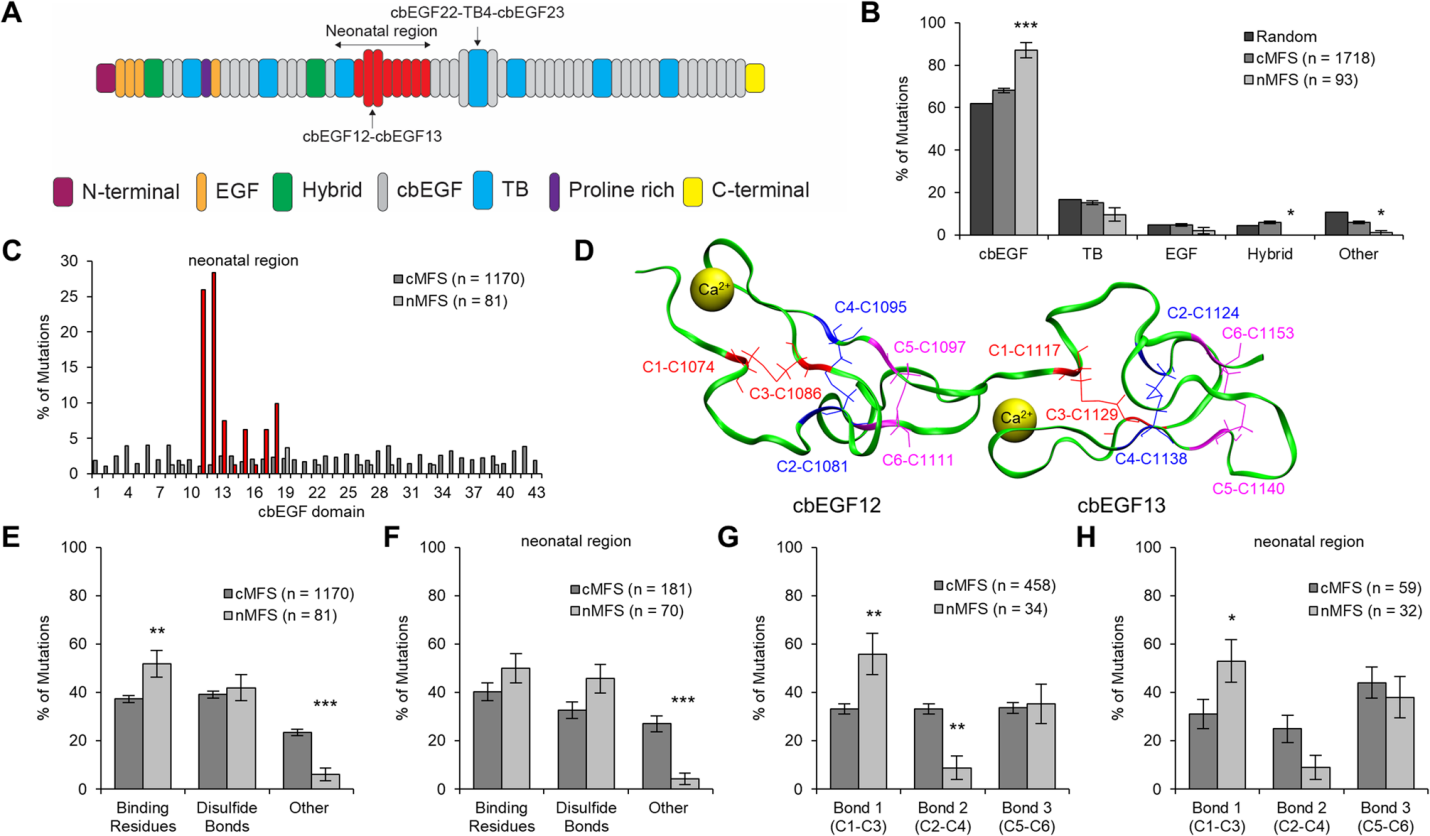
Fibrillin-1 mutations associated with classical MFS (cMFS) and neonatal MFS (nMFS) exhibit distinct cbEGF domain disulfide bond mutation frequency. (**A**) Domain organization of human fibrillin-1: EGF is epidermal growth factor-like domain, cbEGF is calcium binding EGF domain, TB is transforming growth factor beta (TGFB) binding protein-like domain. Neonatal region cbEGF domains shown in red. (**B**) Mutations by domain type (note: random assumes a uniform distribution). (**C**) Mutations by cbEGF domain. cMFS mutations are dark gray. nMFS mutations are light gray. Red bars correspond to nMFS mutations in neonatal region (cbEGF11-cbEGF18). (**D**) NMR structure of cbEGF12-cbEGF13 (PDB ID: 1LMJ) with disulfide bonds 1 (C1-C3; red), 2 (C2-C4; blue), and 3 (C5-C6; magenta) indicated. (**E**,**F**) cbEGF mutations by residue type for full protein and neonatal region, respectively. (**G**,**H**) cbEGF disulfide bond mutations for full protein and neonatal region, respectively. All mutations were acquired from the UMD-FBN1 Mutations Database (https://www.umd.be/FBN1). Error bars represent standard error (SE). Statistical comparisons made between cMFS and nMFS using a Chi-squared test. * p < 0.05, ** p < 0.01, *** p < 0.001. See Table S2 for details on case inclusion and exclusion.

Given the prominent role calcium plays in fibrillin-1 cbEGF domains, we next wanted to evaluate whether potential changes in cbEGF domain calcium binding might underlie phenotypic differences observed between distinct MFS mutations (i.e. cMFS vs nMFS). To identify mutations of interest, we first analyzed the UMD-FBN1 mutations database^31^ and identified 1,811 mutations that met inclusion criteria, with 1,718 categorized as cMFS and 93 categorized as nMFS (Table S2). Next, cMFS and nMFS mutations were grouped based on the domain organization of fibrillin-1 (Fig. 4A). These data confirmed greater probability of finding MFS mutations in cbEGF domains^32^ (Fig. 4B). We further showed that nMFS mutations are significantly more likely to affect cbEGF domains compared to cMFS mutations (87% vs 68%; p < 0.001), findings consistent with previous studies^32^. Focusing on the neonatal region^3,10^, 86% of reported nMFS cbEGF domain mutations occurred in cbEGF domains 11–18, with cbEGF12 being the most affected, whereas cMFS mutations were nearly uniformly distributed (Fig. 4C). These findings imply regional differences in cbEGF domains are important. This is interesting given that cbEGF domains throughout fibrillin-1 exhibit highly similar structure (Fig. 4D) independent of being in the neonatal region^33^.

To assess potential effects of mutations on cbEGF domain function, we next classified mutations as: (1) calcium binding residues; (2) disulfide bond forming cysteine residues; and (3) other residues. Consistent with previous studies, we found mutations were most likely to affect calcium binding residues and disulfide bonds (Fig. 4E). However, unlike previous reports that grouped calcium binding and disulfide bond forming cysteine residue mutations together^32^, we showed the greatest difference is prevalence of mutations in calcium binding residues in nMFS and the significant contribution of “other” mutations to cMFS (Fig. 4E). This remained true when focusing on the neonatal region, although the difference between cMFS and nMFS lost statistical significance for calcium binding residues (Fig. 4F). Nonetheless, the high specificity of calcium binding residue and cysteine residue mutations in conjunction with low occurrence of “other” mutations suggests that nMFS and cMFS mutations have different effects on cbEGF domain function.

Despite disulfide bond forming cysteine residues mutations not showing a significant difference between nMFS and cMFS for the whole protein (42% vs 39%; p = 0.614), the difference trended upward when focusing on the neonatal region (46% vs 33%; p = 0.053). We probed this correlation further by grouping cysteine residue mutations based on the conserved disulfide bond configuration (e.g. C1–C3, C2–C4, C5–C6) found in cbEGF domains. Our results show that nMFS mutations are significantly more likely to affect disulfide bond 1 (C1–C3; 56% vs 33%; p = 0.007) and significantly less likely to affect disulfide bond 2 (C2–C4; 9% vs 33%; p = 0.003) compared to cMFS (Fig. 4G). Disulfide bond 3 was equally affected (C5–C6; 35% vs 34%; p = 0.843). This pattern was preserved when focusing on the neonatal region (Fig. 4H), although the difference between nMFS and cMFS in disulfide bond 2 lost statistical significance (9% vs 25%; p = 0.066). While the relative importance of specific cbEGF domain disulfide bonds has been discussed in the literature, the observation was made in the context of ectopia lentis^34,35^. To our knowledge, this pattern of cbEGF domain disulfide bond mutations has not been explicitly reported in the context of cMFS vs nMFS.

### Disulfide bond mutations disrupt cbEGF domain mechanosensitive calcium binding

Given that cbEGF domain calcium binding dynamics, conformation, and mechanical stress all appear interrelated and the potential for disulfide bonds to stabilize cbEGF domains, probing the observation that nMFS is more strongly associated with mutations in disulfide bond 1 and less strongly associated with mutations in disulfide bond 2 became our primary interest. For cbEGF12-cbEGF13, approximately two-thirds of these disulfide bond mutations were C- > R (32.3%) or C- > Y (32.3%) missense mutations (Table S3). Because simulating all reported mutations was not computationally feasible, we decided to focus on four C- > R mutations (e.g. C1086R, C1111R, C1117R, C1138R). C- > R mutations were chosen to eliminate potential confounding effects caused by comparing different amino acid substitutions (e.g. C- > Y) and because C- > R mutations most closely matched the distinct mutation pattern observed.

#### Disulfide bond 1 serves as the primary fulcrum through which cbEGF domain calcium-binding pockets unfold

**Figure 5:**
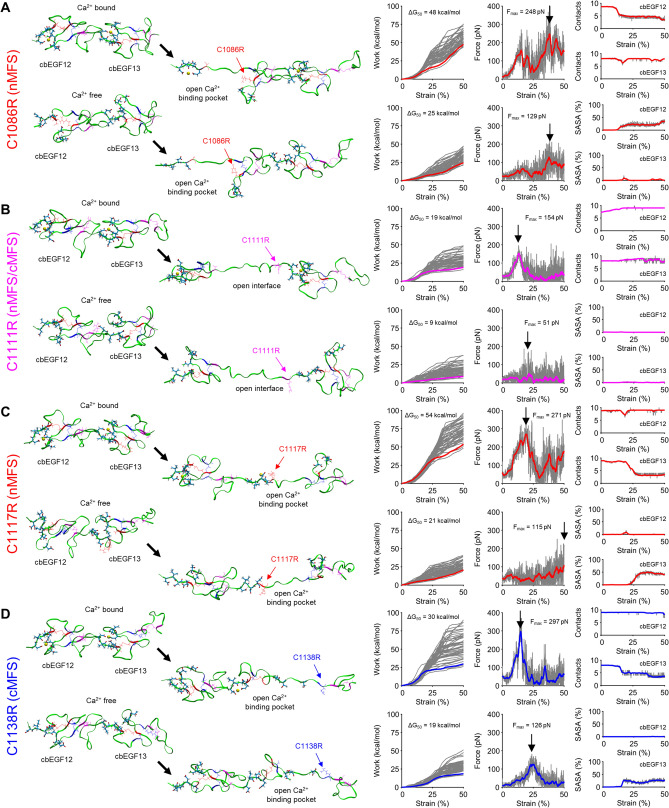
MFS disulfide bond mutations uniquely modulate cbEGF domain stiffness and calcium binding dynamics. (**A**) nMFS mutation C1086R removes disulfide bond 1 in cbEGF12. (**B**) nMFS/cMFS mutation C1111R removes disulfide bond 3 in cbEGF12. (**C**) nMFS mutation C1117R removes disulfide bond 1 in cbEFG13. (**D**) cMFS mutation C1138R removes disulfide bone 2 in cbEFG12. Left side: 0% and 50% strained structures with (top) and without (bottom) calcium, respectively. Right side: Work profiles (n = 50) with Jarzynski average (black line) with free energy change (ΔG_50_), Force profiles with peak force indicated; Left side: Calcium-oxygen contacts and calcium solvent accessible surface area (SASA) as functions a strain.

nMFS mutations C1086R and C1117R eliminated disulfide bond 1 in cbEGF12 and cbEGF13, respectively (Fig. 5A,C). Mutations in cbEGF12 and cbEGF13 were separately investigated to compare the effects of cbEGF-cbEGF inter-domain stabilization. Both mutations resulted in significant unfolding of their respective cbEGF domains beginning between 10–20% strain, with markedly enhanced displacement of β-hairpin residues compared to wild type (Fig. 5A,C; Video S5, Video S6, Video S7, Video S8). This occurred in both cbEGF12 and cbEGF13 due to unfolding of the coiled residues between the first cysteine residues of disulfide bonds 1 and 2, 1074-CRISPDLC-1081 and 1117-CQRDPLLC-1124, respectively. Interestingly, ∆G_50_ values with calcium were relatively unchanged (48 kcal/mol and 54 kcal/mol vs 51 kcal/mol), while ∆G_50_ values without calcium decreased by ~ 36% (25 kcal/mol and 21 kcal/mol vs 36 kcal/mol). Rupture forces were similarly affected (Fig. 5A,C). Furthermore, C1117R resulted in significant unfolding of cbEGF13 despite presence of an inter-domain interface, which simulations of wild-type protein suggested would stabilize cbEGF13. Together, these findings demonstrate calcium-dependent cbEGF domain stability is highly dependent on disulfide bond 1. Loss of disulfide bond 1 allowed cbEGF domain calcium binding β-hairpins to displace further under stress and straightened normally coiled regions, which together resulted in decreased calcium contacts and increased calcium SASA. As with wild type, stress-free equilibrium calculations alone were not sufficient to capture these dynamic effects and appeared to over-predict the stiffening role of calcium (~ 80% vs ~ 55% stiffness decrease; Table S1 and Fig. 5A,C). Overall, these results indicate disulfide bond 1 mutations profoundly decrease cbEGF domain calcium affinity and structural stability, and these effects are potentiated by mechanical stress.

#### Disulfide bond 2 maintains β-hairpin conformation under stress

cMFS mutation C1138R eliminated cbEGF13 disulfide bond 2, which markedly altered cbEGF13 domain unfolding under stress (Fig. 5D). Compared to wild-type protein, ∆G_50_ of C1138R was decreased by 41% (30 kcal/mol vs 51 kcal/mol) with bound calcium and 47% (19 kcal/mol vs 36 kcal/mol) without calcium (Fig. 5D). A primary peak rupture force of 297 pN at 15% strain was observed when calcium was bound and the rupture event coincided with breakage of the C-terminal β-hairpin (Video S9). As the β-hairpin unfolded, a subset of residues remained in partial contact with calcium until complete disassociation at 35% strain, which coincided with the second minor peak (Fig. 5D). As expected, decreased calcium contacts and increased calcium SASA where observed for the mutated domain (i.e. cbEGF13). As calcium dissociated, cbEGF12 domain flexibility increased and the 13 residues between C1124 and R1138 (~ 4.6 nm) straightened, which resulted in decreased tension compared to wild-type protein. Without calcium, a similar unfolding pattern was observed, except that the peak force decreased to 126 pN and shifted rightward to 25% strain, likely due to increased cbEGF12 flexibility upon calcium removal (Video S10). Together, these results demonstrate that disulfide bond 2 is crucial for maintaining tension in cbEGF domains by stabilizing β-hairpin conformation, particularly under moderate to high mechanical stress when calcium is not bound to the β-hairpin.

#### Disulfide bond 3 stabilizes calcium-dependent cbEGF-cbEGF inter-domain interfaces

nMFS/cMFS mutation C1111R eliminated cbEGF12 disulfide bond 3, which markedly altered cbEGF12-cbEGF13 unfolding under stress (Fig. 5B). Compared to wild-type protein, ∆G_50_ of C1111R decreased by 63% (19 kcal/mol vs 51 kcal/mol) with bound calcium and 75% (9 kcal/mol vs 36 kcal/mol) without calcium (Fig. 5B). With calcium bound, interface rupture was observed at ~ 13% strain and a peak rupture force of 154 pN. Both measures were markedly decreased in C1111R compared to wild-type protein, demonstrating increased flexibility in cbEGF domain mutants lacking disulfide bond 3. We further showed that without calcium, stability of the cbEGF-cbEGF inter-domain interface was greatly compromised in C1111R (Fig. 5B). Together, these results demonstrate that disulfide bond 3 serves as an important stress-bearing link in cbEGF-cbEGF inter-domain interfaces. Interestingly, despite this crucial biomechanical role, loss of disulfide bond 3 does not appear to directly affect calcium binding. Calcium contacts and SASA remained mostly unchanged from 0–50% strain (Fig. 5B). The marginal increase in calcium contacts observed with strain likely reflects reduction in calcium binding pocket stress due to increased inter-domain flexibility following interface rupture. Indeed, C1111R was the only mutant investigated to show significantly reduced equilibrium stiffness compared to wild type (holo: 2062 ± 2298 pN vs 4306 ± 2342 pN, p = 0.044; apo: 693 ± 275 pN vs 1691 ± 1208 pN, p = 0.020).

## Discussion

Mechanosensitive proteins—including stretch-activated ion channels (e.g. TRP, TREK, Piezo)^36^, cytoplasmic integrin-associated proteins (e.g. talin)^37^, and nuclear envelope proteins^38^—are fundamental to mechanobiology. As reflected by these examples, the study of mechanosensitivity has traditionally been focused on cells. Nevertheless, extracellular matrix, which bears most tissue forces, also exhibits mechanosensitive properties. For example, stretch-induced conformational changes in fibronectin expose cryptic integrin binding domains that have pleiotropic effects on local cells^39,40^ and tension in collagen fibers reduces proteolytic susceptibility, suggesting molecular strain plays an essential role in normal extracellular matrix structure and function^41–43^.

Here, we extend the idea that extracellular matrix is mechanosensitive by demonstrating through computational modeling the combined effects of mechanical stress and MFS-associated cysteine mutations on fibrillin-1 cbEGF domain calcium binding dynamics. The premise of these studies was established in previous studies demonstrating biophysical consequences of calcium removal^14–16^. However, these previous works that examined effects of fibrillin-1 mutations at stress-free equilibrium failed to account for the important variable of mechanical stress, which is a key factor in normal matrix function. Through interrogating these models with non-equilibrium dynamics induced by mechanical stress, our results shift paradigms in connective tissue biology by reframing fibrillin-1 as an intrinsically mechanosensitive protein and provide a unique perspective of MFS as a disorder of matrix mechanosensitivity rather than a cell-focused disorder of integrin binding or intracellular signaling^44–46^.

### cbEGF domains are stress responsive calcium actuated switches

**Figure 6:**
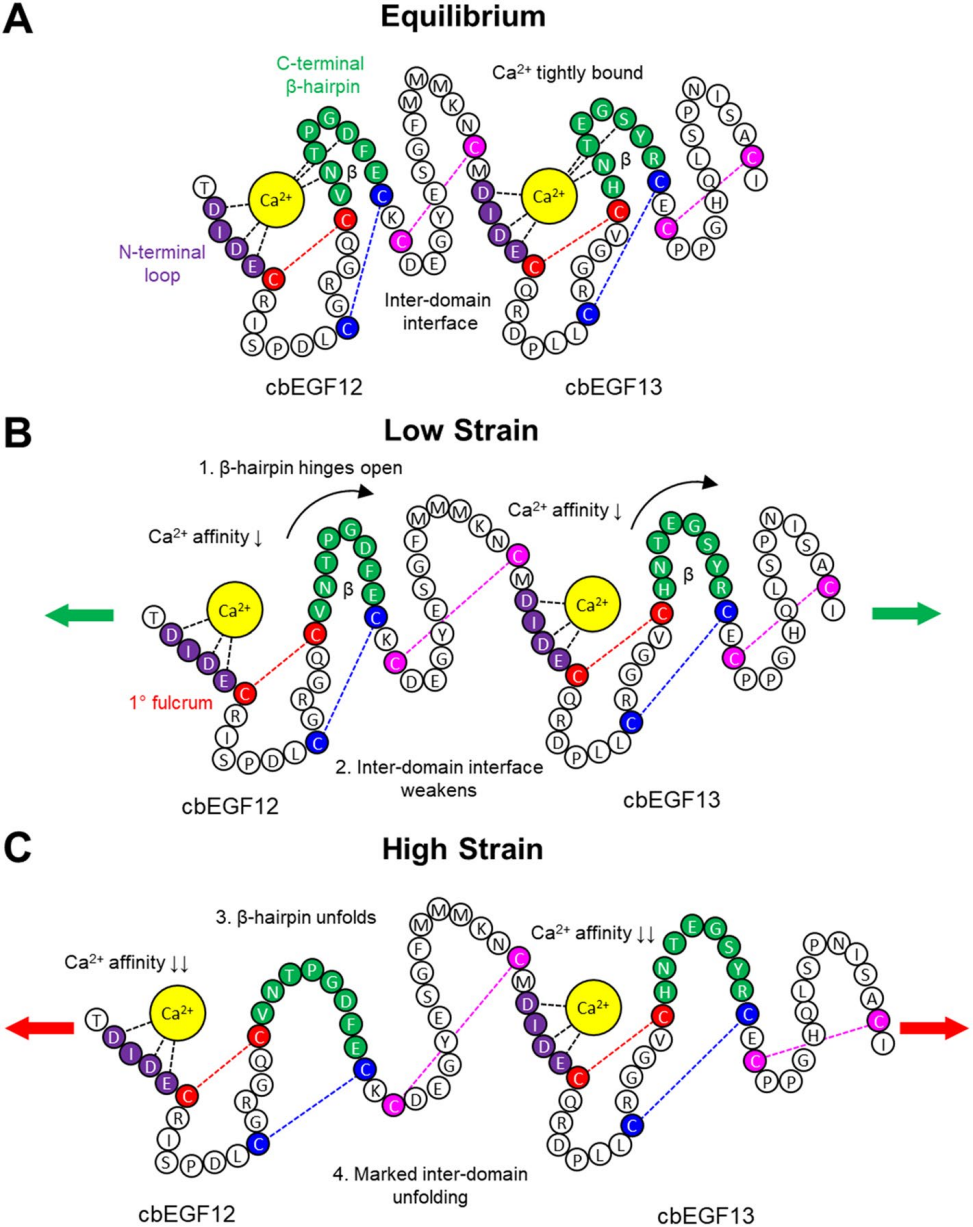
Biophysical model of cbEGF domain mechanosensitive calcium binding. (**A**) Equilibrium structure without external forces exhibits robust calcium-dependent inter-domain interfaces. (**B**) Low strain causes β-hairpin to switch away from calcium leading to decrease calcium affinity and weakened inter-domain interfaces. Calcium dominates low strain elasticity. (**C**) High strain causes marked β-hairpin and inter-domain unfolding. Disulfide bonds and backbone residues dominate high strain elasticity. Note: cbEGF12 shows enhanced unfolding compared to cbEGF13 to illustrate the stabilizing effect of inter-domain interfaces.

Each cbEGF domain calcium binding pocket is composed of two parts, an N-terminal loop and a C-terminal β-hairpin. At stress-free equilibrium, calcium coordinates with key binding residues to maintain the pocket in a closed conformation (Fig. 6A). The closed conformation stabilizes cbEGF domains and favors robust cbEGF-cbEGF inter-domain interfaces, which further stabilizes calcium binding. Calcium-dependent stabilization is thus responsible for stiffening and extending tandem cbEGF domain repeats into rigid rod-like structures^11^. As cbEGF domains are mechanically stretched, the β-hairpin unbinds calcium and pivots away from the N-terminal loop (Fig. 6B). Progressive unbinding of calcium contacts reduces cbEGF domain stability resulting in structural rearrangements characterized by marked inter- and intra-domain unfolding. The net result is increased cbEGF domain flexibility and decreased calcium affinity (Fig. 6C). A potential consequence of calcium unbinding and inter-domain unfolding is destabilization of adjacent cbEGF domains. Interactions between adjacent cbEGF domains, combined with distinct calcium binding affinities, could provide a mechanism for cooperative tuning of stiffness regionally or propagation of changes in flexibility globally. Our model thus describes calcium unbinding as a switch-like event that occurs in response to mechanical stress, wherein calcium affinity depends on cbEGF domain tension.

### Disulfide bonds orchestrate calcium actuated switching

Although previous studies recognized the importance of disulfide bonds in stabilizing cbEGF domains, our simulations reveal crucial and distinct roles for each bond in orchestrating stress-induced calcium-dependent unfolding. Here, disulfide bond 1 serves as the primary fulcrum through which switching of the calcium binding pocket occurs and limits displacement of the unbound β-hairpin under stress. In support of this action, disulfide bond 2 stabilizes the β-hairpin to prevent complete unfolding, thus preserving the structure of the β-hairpin when the switch is actuated. And finally, disulfide bond 3, which resides furthest from the binding pocket, stabilizes the calcium-dependent interface between adjacent cbEGF domains; this prevents the two domains from separating, thus concentrating stress to the binding pocket to actuate calcium-dependent β-hairpin switching. Disulfide bonds therefore ensure the mechanical response of cbEGF domains is actuated through mechanosensitive calcium binding dynamics. Although this investigation specifically assessed unfolding dynamics, we speculate our model encompasses elasticity more generally by allowing robust calcium-dependent refolding. Indeed, ordered unfolding and refolding of mechanically stretched cbEGF domains triggered by conserved mechanosensitive calcium binding at specific regions in fibrillin-1 could constitute a fundamental feature of microfibril mechanobiology. Our model thus generalizes mechanosensitive calcium binding as an intrinsic biophysical property inherent to cbEGF domains. This reframes previously discounted cbEGF domain repeats, which compromise most of fibrillin-1, as essential mechanosensitive elements.

### Different mechanisms compensate for low and high strain

Calcium-dependent cbEGF domain elasticity is intriguing given that a calcium-dependent recoil mechanism was previously attributed to TB-cbEGF and hybrid-cbEGF interfaces^29^. Indeed, previous studies discounted tandem cbEGF-cbEGF domain repeats as significant contributors to fibrillin-1 extensibility based on the observations that cbEGF domains are stiffer and more extended when calcium is bound. While our baseline simulations confirmed these equilibrium observations, SMD simulations revealed cbEGF domain stiffness decreases under strain due to mechanosensitive calcium unbinding. Moreover, SMD simulations revealed cbEGF12-cbEGF13 and TB4-cbEGF23 were similarly stiff at low strain, with these two domain pairs exhibiting calcium-dependent and calcium-independent interfaces, respectively. Although the finding of calcium independence at the TB4-cbEGF23 inter-domain interface appears in conflict with previous reports, it is consistent with observations that crystal structures of calcium bound and calcium free TB4-cbEGF23 are indistinguishable^24^. Although previous studies speculated removal of calcium would result in loss of interface stability^24^, crystal structures were consistent with calcium independence as shown by our SMD results. Overall, these results support a crucial contribution of calcium-dependent stiffness in tandem cbEGF-cbEGF domain repeats to fibrillin-1 flexibility at low strain insufficient to rupture calcium-independent TB-cbEGF domain interfaces.

The situation appears different at high strain. SMD simulations confirm TB-cbEGF interfaces are more flexible at high strain following inter-domain interface rupture due to the predicted flexible linker. Thus, although tandem cbEGF domains and TB-cbEGF domains appear similarly stiff at low strain, rupture of inter-domain interfaces at high strain is likely to initiate at discrete positions in the protein where interfaces are weakest. This implies low strain calcium-dependent unfolding could be at play in tandem cbEGF domain repeats either before forces are sufficient to rupture calcium-independent TB-cbEGF domain interfaces or after TB-cbEGF interfaces are already extended. Thus, while TB-cbEGF domains interfaces likely still play an essential role in fibrillin-1 extensibility as previously proposed, our results strongly suggest tandem cbEGF domains, the most prominent domain structure in fibrillin-1, have high potential to contribute significantly to fibrillin-1 flexibility while being resilient enough to stretch reversibly under a wide range of physiologic forces due to their relative stiffness. Overall, these dynamics may explain more recent observations that challenge the rigid rod-like conformation of tandem cbEGF repeats originally proposed^47,48^.

### Relevance to matrix remodeling

Until now, we have only considered cbEGF domain mechanosensitive calcium binding in the context of modulating fibrillin-1 structure and stiffness. However, the structural dynamics that allow ordered cbEGF domain unfolding and refolding might have crucial biological roles in regulated elastic tissue remodeling. For example, transforming growth factor beta (TGFB), a key inducer of matrix remodeling, is sequestered in matrix by the latent TGFB binding protein (LTBP) component of large latent TGFB complexes (LLCs). These LLCs have been shown to bind fibrillin-1^49,50^. Moreover, LLCs are composed of cbEGF repeats similar to fibrillin-1^51^. Thus, physical interactions between fibrillin-1 and LLCs could function as stretch-sensitive hubs^45^, with mechanosensitive calcium binding acting as the switch-like event that modulates extracellular TGFB sequestration. Indeed, matrix strain has been implicated as an important factor in TGFB activation and tissue under stress have been shown to have increased TGFB bioavailability^52^. Microfibril strain or cell adhesion forces applied to LTBPs could thus promote localized changes in protein structure, controlled via calcium switching, to regulate TGFB bioavailability and induces matrix remodeling. Furthermore, proteolytic degradation is an obligatory step in matrix remodeling. Previous studies identified proteolytic cleavage sites in fibrillin-1 between cbEGF domain disulfide bonds and demonstrated that bound calcium protects fibrillin-1 from proteolytic degradation^17^. Our observations of strain induced unfolding of cbEGF domains suggest that mechanical stress may expose proteolytic cleavage sites in fibrillin-1 to increase the rate of strained microfibril degradation.

Together, dynamic LLC binding and proteolytic cleavage sites could represent integrated “smart” features that regulate matrix remodeling in a mechanosensitive manner. Our SMD studies suggest that at low strain, neither module would be readily activated. However, at increasing strain, TB domain interfaces could rupture, followed by cbEGF domain unfolding. Thus, TGFB release may precede exposure of proteolytic cleavage sites to coordinate the process of matrix remodeling. Implicit in this model is a common mechanism that promotes TGFB dependent matrix reinforcement at moderate strain and facilitates preferential targeting of proteolytic enzymes to remove over-stretched microfibrils. Although not explicitly addressed in this study, we propose the sensitivity of these “smart” features could be fine-tuned by matrix pre-stress generated by tissue growth, osmotic pressure, and baseline physiologic forces (e.g. arterial blood pressure). This could leave the state of TGFB sequestration and cbEGF domain calcium binding delicately balanced on a knife-edge to maintain elastic tissue homeostasis.

### Biophysical aspects of Marfan syndrome phenotypes

A major conceptual challenge is how the large number of single amino acid substitutions (i.e. missense mutations) distributed throughout the approximately 350 kDa fibrillin-1 molecule each results in the phenotypic presentation of MFS. Extensive studies have identified: (1) dominant-negative effects of mutations that interfere with microfibril structure^34,53^ and intracellular trafficking or extracellular secretion^54,55^; and (2) haploinsufficiency resulting from gene deletions^56^, defective alternative splicing^57^, and increased proteolytic turnover^58^. The net result is matrix deficient in normal microfibrils. However, this does not explain development of phenotypes when “normal” levels of microfibrils are initially present.

Mechanosensitive calcium binding adds a new dimension to MFS disease models. Our model predicts mutations that perturb calcium binding, alter calcium binding pocket stress, or affects inter-domain interface stability, would affect fibrillin-1 elasticity and could alter TGFB bioavailability and proteolytic sensitivity, leading to elevated levels of abnormal matrix remodeling. Dominant-negative mutations could thus result from cbEGF domain destabilization and marked calcium unbinding that mimics an over-tensioned state, a constant “on-switch” in extracellular signaling. Conversely, in haploinsufficiency, sparse wild-type microfibrils incorporated into developing matrix could assume true over-stretched “on-switch” states under normal physiological stress. In either case, hyper-activation of stretch-dependent signaling (e.g. TGFB activation) and proteolysis could drive inappropriate matrix remodeling that further exacerbates matrix dysfunction. In fact, the positive feedback intrinsic to this system would result in progressive degeneration that parallels the typical clinical observations of MFS with age. Thus, mechanosensitive calcium binding offers a structural/functional explanation for how localized point mutations could have global effects and suggests the true consequences of many *FBN1* mutations are not fully observable at stress-free equilibrium.

Influence of the nano-mechanical environment on matrix structure/function could thus be a crucial determinant of genotype/phenotype relationships in MFS. Genotype in combination with intrinsic tissue strain could establish critical thresholds that sensitize matrix remodeling to biomechanical demands. Importance of the nano-mechanical environment also provides an attractive explanation for why certain mutations associated with nMFS (e.g. disulfide bond 1 mutations) do not always produce the most severe phenotype of early neonatal lethality. For example, while our simulations reveled disulfide bond 1 operates as the primary fulcrum through which cbEGF domain calcium binding pockets switch open, functional consequence of eliminating disulfide bond 1 depends on mechanical context. Our model posits that mutations may have limited effect on stress-free equilibrium protein structure, but have pronounced, though variable, effects on fibrillin-1 structure and function dependent on local stress. Variability reflects the probability of events occurring that increase matrix remodeling (e.g. TGFB release or activation of proteolytic cleavage) or affect protein biomechanics and transfer stress to unaffected molecules. Frequency and amplitude of stress combine to increase probability of these events via increasing the period cbEGF domains are partially or completely unfolded. In addition, as structural constraints have likely evolved to ensure high fidelity protein refolding during relaxation, frequency and amplitude of stress could also collaborate with genotype to increase probability of incorrect refolding and functional loss of the misfolded molecule. Cooperative unfolding and calcium unbinding could propagate these errors to adjacent domains that, in turn, would increase protein dysfunction. Loss of fibrillin-1 function thus leads to a domino effect that ultimately first affects individual microfibril function and later disrupts tissue function. Thus, mutations that primarily affect fibrillin-1 biomechanics are predicted to stochastically influence fibrillin-1 structure/function, thereby resulting in progressive phenotypes with variable expressivity dependent on the nano-mechanical environment.

## Conclusions

In conclusion, SMD simulations have revealed the biophysical mechanism through which calcium binding dynamics modulates fibrillin-1 cbEGF domain unfolding. We demonstrate that calcium exerts its stabilizing role predominately at low strain, after which the β-hairpin of the calcium-binding pocket switches open, facilitating a decrease in calcium affinity. We show that disulfide bond 1, the most frequently mutated disulfide bond in nMFS, plays a crucial role in limiting β-hairpin displacement, serving as the primary fulcrum through which the calcium binding pocket switches open. Integrating our model with pervious knowledge, we propose cbEGF domain mechanosensitive calcium binding represents an evolutionarily conserved high-limit stretch-sensor for fibrillin-1 microfibrils. Such an integrated “smart” feature could regulate mechanosensitive protein–protein interactions in matrix including TGFB sequestration and microfibril degradation. Mutations causing MFS may therefore over-activate normal microfibril responses to mechanical stress. Such a concept reframes microfibrils as calcium-dependent mechanosensitive hubs directly involved in regulating elastic tissue homeostasis.

## Methods

### UMD-FBN1 mutations database analysis

The UMD-FBN1 mutations database (www.umd.be/FBN1) ^31^ was searched and analyzed using a custom written MATLAB code (R2018a, The MathWorks, Inc., Natick, Massachusetts, US). A total of 3231 mutation records were identified. Mutations from patients with neonatal MFS (nMFS; n = 93) were defined using the keywords “neonatal MFS” or “intantil MFS” for disease type. Mutations from patients with classical MFS (cMFS; n = 1,718) were defined using the keywords “Classical MFS” or “MFS” for disease type. The remaining 1,420 records were excluded because they were of unknown or inapplicable categorization (Table S2). Results were reported as percentage of mutations with statistical comparisons made between nMFS and cMFS using a Chi-squared test. P-values less than 0.05 were considered statistically significant. Random bars in Fig. 4B were based on a random uniform distribution of mutations in the human fibrillin-1 protein sequence, with domain ranges defined per the UNMD-FBN1 mutations database.

### cbEGF12-cbEGF13 and TB4-cbEGF23 domain structures

NMR and X-ray diffraction structures were obtained from the RCSB Protein Databank (PBD; rcsb.org)^59^ for the human fibrillin-1 domains cbEGF12-cbEGF13 (PDB ID: 1LMJ)^13^ and cbEGF22-TB4-cbEGF23 (PDB ID: 1UZJ)^24^, respectively. cbEGF12-cbEGF13 constituted the primary focus of this study. TB4-cbEGF23 was included for comparison. All structures were visualized using Visualize Molecular Dynamics (VMD) 1.9.3^60^.

### Molecular dynamics simulations

All molecular dynamics simulations were performed using the Amber18 and AmberTools19 software packages (AMBER 2018, University of California, San Francisco, California, US). The ff14SB force field^61^ and a non-bond cutoff distance of 12.0 Å was applied using the Particle Mesh Ewald (PME) method with periodic boundary conditions. The SHAKE algorithm was applied for all bonds involving hydrogen, which allowed for a time-step size of 4 fs when combine with hydrogen mass repartitioning (HMR). To model non-covalently bound calcium ions and capture changes in coordination induced by strain, the 12–6 Lennard–Jones non-bonded model included with the ff14SB force field was used. Structures were solvated in TIP3P water with X–Y–Z edge buffer distances of 12 Å × 30 Å × 12 Å (cbEGF12-cbEGF13) or 12 Å × 31 Å × 12 Å (TB4-cbEGF23) and neutralized with sodium counter ions. This resulted in final box dimensions of 53.8 Å × 118.9 Å × 54.4 Å containing 8,573 water molecules for wild-type cbEGF12-cbEGF13 and 60.3 Å × 139.8 Å × 77.7 Å containing 17,234 water molecules for wild-type TB4-cbEGF23. The long axis of the box (i.e. Y axis) was purposefully enlarged to allow for subsequent stretching of the proteins.

### Disulfide bond mutations

Missense mutations were introduced into cbEGF12-cbEGF13 based on our analysis of the UMD-FBN1 mutations database^31^. Residues were swapped using the AmberTools19 sub-program *PDB4amber*. Structures were then reduced using the AmberTools19 sub-program *reduce*^62^. Overlaps caused by the mutation process removed using *tleap*. Supplementary Table S3 shows all cysteine disulfide bond mutations in cbEGF12-cbEGF13 reported in the UMD-FBN1 mutations database^31^. Internal mutations were prioritized to minimize potential boundary effects caused by domain fragments not in the whole protein (i.e. the C-terminal of cbEGF13 should continue with the N-terminal of cbEGF14, not terminate into solvent). For simulations without bound calcium, calcium atoms were deleted from the PDB files prior to equilibration.

### System equilibration

Systems were equilibrated to constant pressure and temperature for subsequent NPT simulations using a multi-step process optimized for the specific proteins in this study. Special attention was given to prevent the axis-aligned proteins from rotating, as the long axis of the protein needed to be maintained in alignment with the long axis of the solvent box for subsequent stretching.

First, systems were minimized over two 10,000 iteration stages, the first of which had a 20 kcal/mol/Å^2^ restraint constant applied to all non-solvent residues. For each stage, the steepest decent method was used for the first 500 iterations before switching to the conjugate gradient method for the remaining 9,500 iterations. This approach was sufficient to converge the system to a local energy minimum and remove any high-energy artifacts caused by the insertion of the mutated residues and solvent molecules.

Next, the system was heated from 0 to 300 K over 0.5 ns and maintained using a Langevin thermostat. 300 K was selected as a standard temperature to enable future biophysical comparisons. Non-solvent residues had a 20 kcal/mol/Å^2^ restraint constant applied to prevent rotation and avoid artifacts from local over-heating. Next, the system was pressurized from 0 to 1 bar over 0.5 ns and maintained using a Monte Carlo barostat. Non-solvent residues had a 20 kcal/mol/Å^2^ restraint constant applied to prevent rotation. Finally, the system was allowed to equilibrate over two 1 ns stages, during the first of which the non-solvent restraints were gradually reduced in five 0.2 ns stages based on the following scheme: (1) t = 0.0–0.2 ns, r = 20 kcal/mol/Å^2^; (2) t = 0.2–0.4 ns, r = 10 kcal/mol/Å^2^; (3) t = 0.4–0.6 ns, r = 5 kcal/mol/Å^2^; (4) t = 0.6–0.8 ns, r = 2.5 kcal/mol/Å^2^; (5) t = 0.8–1.0 ns, r = 1 kcal/mol/Å^2^. During the final 1 ns stage, the system was simulated with a 1 kcal/mol/Å^2^ restraint applied only to the N-terminal nitrogen and C-terminal carbon atoms to prevent rotation.

### Equilibrium length and straightness

Equilibrium length and straightness were calculated with and without bound calcium based on 10 independent 40 ns trajectories conducted without restraints following equilibration (note: the first 20 ns was discarded). For cbEGF12-cbEGF13, length was defined as two times the Euclidean distance between the center of mass of cbEGF12 (e.g. D1070-M1112) and cbEGF13 (e.g. D1113-I1154). Distance between the N-terminal nitrogen and C-terminal carbon was not used because these “free-ends” do not reflect the native protein. Straightness was defined as the negative dot product of the normalized vectors drawn from the center of mass of cbEGF12-cbEGF13 (e.g. D1070-I1154) to the corresponding centers of mass of cbEGF12 and cbEGF13. Stiffness was calculated based on the methods by Adamovic et al.^22^. Here, the apparent stiffness modulus was calculated as.

1 $$K=\frac{kT}{\langle {({L}_{o}-L)}^{2}\rangle }L$$

where $k$ is the Boltzmann constant, $T$ is absolute temperature, ${L}_{o}$ is the instantaneous length between cbEGF12 and cbEGF13, and $L$ is the mean length (note: brackets <  > denote average). Statistical comparisons were made using a two-tailed t-test and two-sample F-test for means and standard deviations, respectively. Each trajectory average following equilibration was considered an independent sample (n = 10).

### Steered molecular dynamics (SMD)

SMD simulations were performed at constant pulling speeds to calculate non-equilibrium work profiles and generate strained structures (Fig. S1). To achieve this, equilibrated structures were first restrained to the equilibrium length of the calcium bound wild-type protein (e.g. 56 nm for cbEGF12-cbEGF13) at the N- and C-terminals using a restraint constant of 100 kcal/mol/Å^2^. Following an additional 20 ns period of restraint, 100 snapshots were acquired at 2 ns intervals to generate independent initial structures for SMD.

Next, each snapshot was randomized with initial velocities to compute independent SMD trajectories. The N-terminal nitrogen was left restrained and a steering potential was then applied between the N-terminal nitrogen and C-terminal carbon in the aligned axis using the *jar* function integrated within Amber18. A spring constant of 7.4 kcal/mol/Å^2^ based on previous studies to satisfy the stiff spring assumption^21^ was used and found to work well; spring constants of 1 kcal/mol/Å^2^ and 100 kcal/mol/Å^2^ showed minimal differences. A total stretch distance of 30 Å was simulated for cbEGF12-cbEGF13 (i.e. 300 ns per trajectory) and 50 Å for TB4-cbEGF23 (i.e. 500 ns per trajectory).

Changes in free energy along the stretching axis were used to construct potential of mean force (PMF) profiles. To achieve this, we employed the Jarzynski relation,

2 $${e}^{-\Delta \mathrm{G}/kT}=<{e}^{-W/kT}>$$

where the exponential change in Gibbs free energy ($\Delta \mathrm{G}$) is related to the average exponential non-equilibrium work ($W$) obtained from the NPT ensemble sampled via SMD (note: brackets <  > represent average, $k$ is the Boltzmann constant, and $T$ is absolute temperature). Force profiles were then calculated as the distance derivative of the PMF profile. A smoothed work profile was obtained using a moving average with a window size of 10 ns.

Pull speeds of 100 Å/ns, 10 Å/ns, 1 Å/ns, and 0.1 Å/ns with sampling numbers of 10, 25, 50, and 100 were tested to assure PMF convergence, which depends on sufficient sampling of low energy paths (Fig. S2). The pull speed of 0.1 A/ns with 50 samples was found to be adequate and computationally practical. Additional validations included: (1) restraining the C-terminal carbon and pulling the N-terminal nitrogen, which showed no differences; (2) using a non-bond cutoff distance of 8 Å instead of 12 Å, which showed negligible differences; and (3) starting all SMD trajectories from the same equilibrated structure, which showed no differences.

### Calcium contacts and solvent accessible surface area (SASA)

Calcium contacts were determined using the amber18 subprogram cpptraj function *nativecontacts* with a defined distance of < 4.0 Å. Calcium solvent accessible surface area (SASA) was calculated using visualize molecular dynamics (VMD) with a sphere size of 1.4 Å and normalized to the surface area of calcium (i.e. 100% ≡ 105.7 Å^2^). Per the Jarzynski equality, a weighted average was used across all samples to calculate calcium contacts and SASA.

3 $$\stackrel{-}{X}=\sum {w}_{i}{X}_{i}$$

where $\stackrel{-}{X}$ is the averaged variable of interest (e.g. SASA or number of calcium contacts), ${X}_{i}$ is the value from trajectory $i$, and ${w}_{i}$ is the weight associated with trajectory $i$ of form.

4 $${w}_{i}=\frac{{e}^{-\frac{{W}_{i}}{kT}}}{\sum {e}^{-\frac{{W}_{i}}{kT}}}$$

where ${W}_{i}$ is the work of trajectory $i$, $k$ is the Boltzmann constant, and $T$ is absolute temperature.

### Computations

Simulations were performed on a computing cluster using a mixture of Nvidia Tesla V100 and P100 GPUs and the GPU implementation of Amber18 (pmemd.cuda). For stability reasons, select equilibration steps (e.g. pressurization) were performed using Intel Xeon E5-2670 CPUs and the MPI implementation of Amber18 (pmemd.mpi). Overall, this study totaled ~ 250 µs of simulation time which required ~ 20,000 GPU-hours.

## Supplementary information

Supplementary Information

Supplementary Video S1

Supplementary Video S2

Supplementary Video S3

Supplementary Video S4

Supplementary Video S5

Supplementary Video S6

Supplementary Video S7

Supplementary Video S8

Supplementary Video S9

Supplementary Video S10

Supplementary Video S11

Supplementary Video S12
