# Supplementary material for: Steered molecular dynamic simulations reveal Marfan syndrome mutations disrupt fibrillin-1 cbEGF domain mechanosensitive calcium binding
Source: Sci Rep. 2020 Oct 8;10:16844. doi: 10.1038/s41598-020-73969-2 (PMC7545174; doi:10.1038/s41598-020-73969-2)
Supplement: Supplementary file 1 — Supplementary Information [file 41598_2020_73969_MOESM1_ESM.pdf]

Supplementary Information

Steered molecular dynamic simulations reveal Marfan syndrome mutations disrupt fibrillin-1 cbEGF domain mechanosensitive calcium binding

Stephen J. Haller[1], Adrian E. Roitberg[2], Andrew Dudley[1,*]

[1] Department of Genetics, Cell Biology and Anatomy, University of Nebraska Medical Center, Omaha, NE, USA

[2] Department of Chemistry, University of Florida, Gainesville, FL, USA

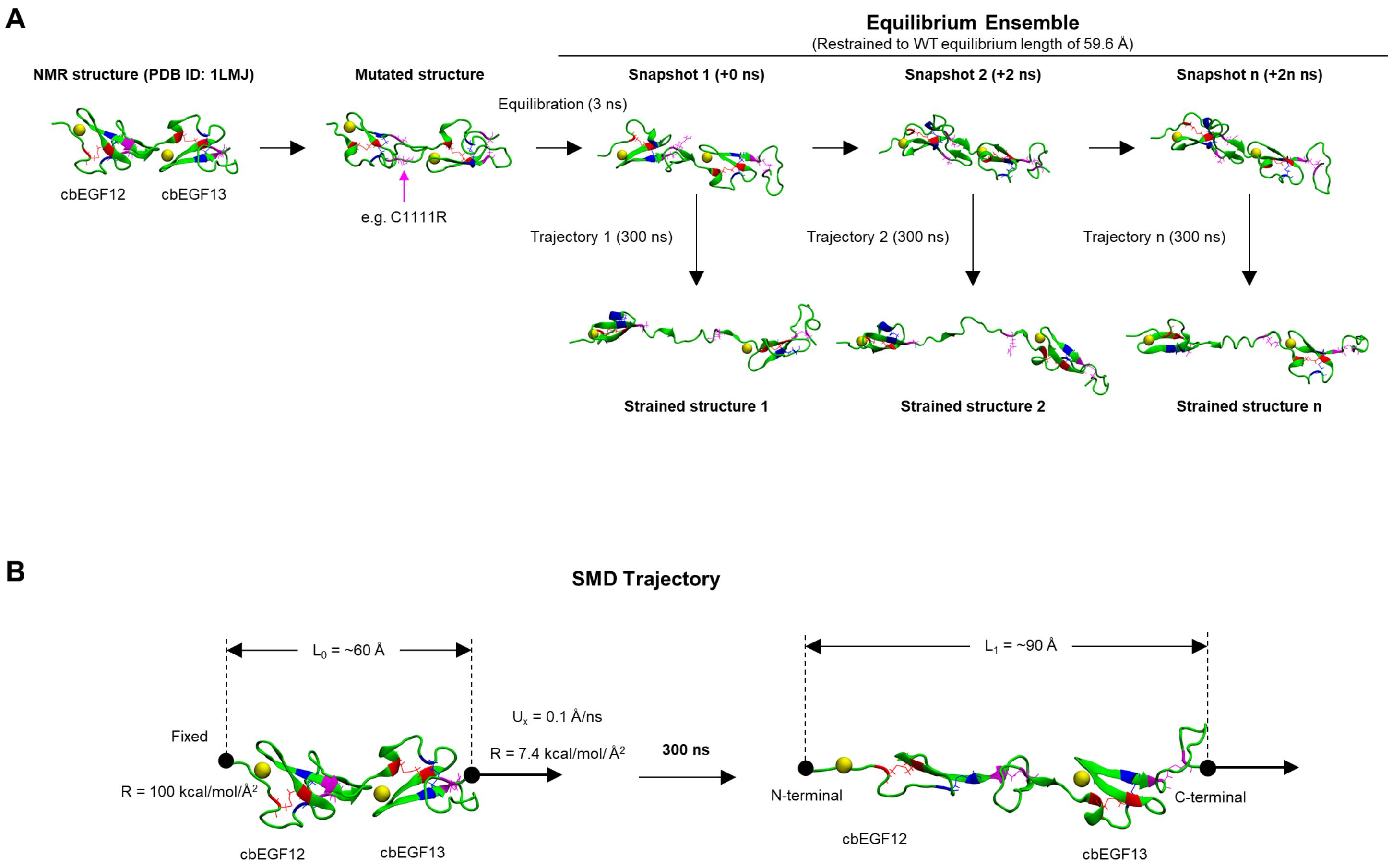

Figure S1. Steered molecular dynamics (SMD) workflow. (A) NMR structure of cbEGF12-cbEGF13 (PDB ID: ILMJ) was mutated and equilibrated (Figure S1). Snapshots (n = 100) were sampled from the NPT ensemble 2 ns apart to initialize independent SMD trajectories. Structures were restrained to wildtype equilibrium length (59.6 Å) for comparable starting lengths. (B) Each SMD trajectory pulled from ~60 Å to ~90 Å (50% strain) over 300 ns (velocity = 0.1 Å/ns) using a spring constant of 7.4 kcal/mol/Å^2.

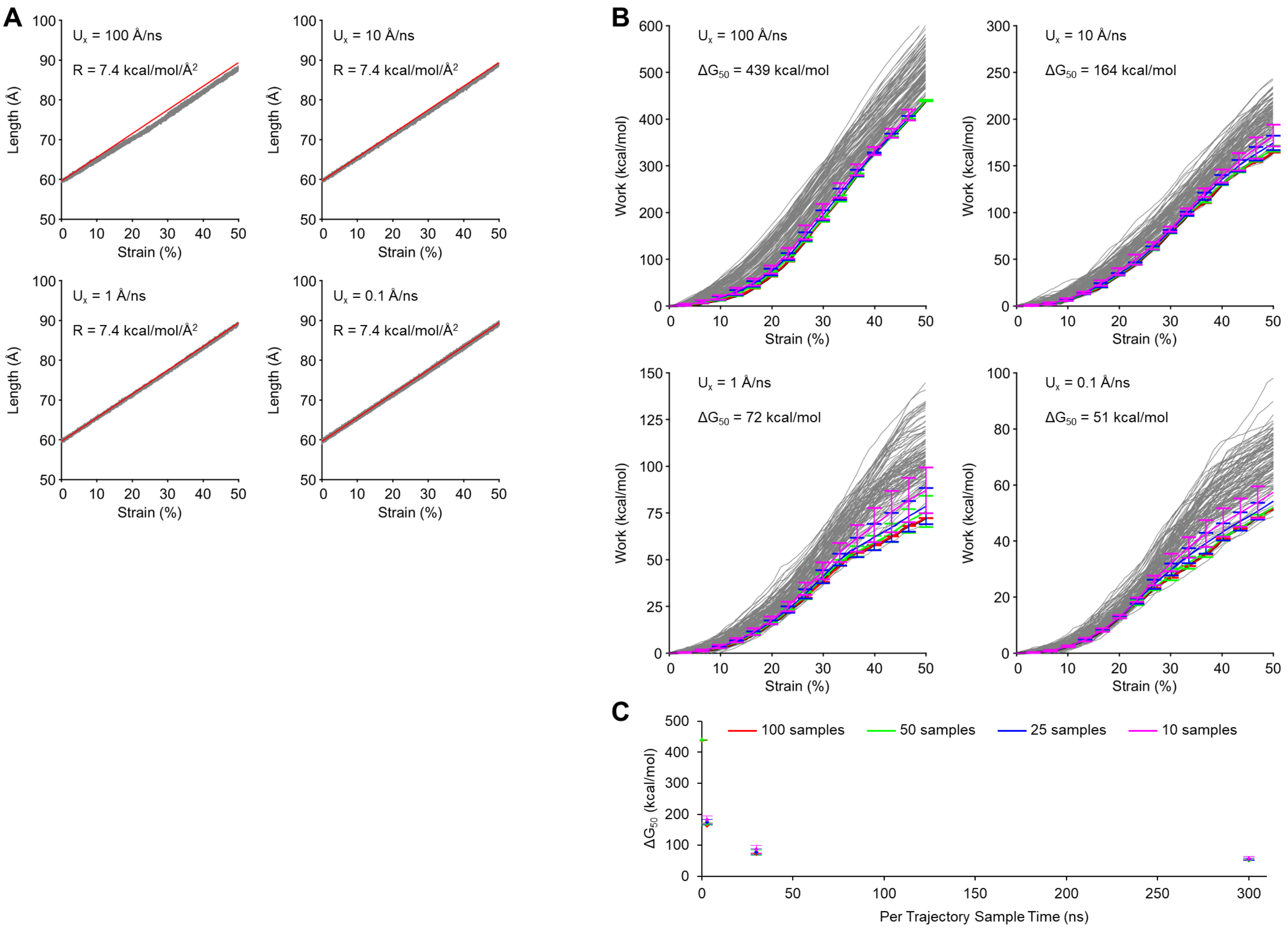

Figure S2. SMD simulation validations for wildtype calcium bound cbEGF12-cbEGF13. (A) Set distance profile (red) versus actual distance profiles (n = 100) at pulling speeds of 100 Å/ns, 10 Å/ns, 1 Å/ns, and 0.1 Å/ns. (B) Work profiles (n = 100) with Jarzynski averages calculated via bootstrapping with sample counts of 100 (red), 50 (green), 25 (blue), and 10 (magenta). Error bars represent standard deviations of 10 independent random samplings of respective counts. (**C**) Free energy change from 0-50% strain (ΔG_{50}) for different bootstrapping schemes over different pull speeds demonstrate convergence at 0.1 Å/ns with 50 samples (e.g. 300 ns per trajectory = 30 Å / 0.1 Å/ns).

Table S1. Equilibrium stiffness calculations

	$\langle (L_o - L)^2 \rangle$ *(Å^2)*	*Length L (Å)*	*Apparent modulus (pN)*	*% change*	*p-value*
Calcium bound (holo)					
WT	0.39 ± 0.25	29.66 ± 1.01	4306 ± 2342		
C1086R	0.24 ± 0.19	29.45 ± 1.46	7038 ± 3437		
C1111R	2.44 ± 4.40	29.52 ± 1.16	2062 ± 2298		
C1117R	0.36 ± 0.40	28.43 ± 0.36	4799 ± 1781		
C1138R	0.63 ± 0.45	29.26 ± 1.18	3321 ± 2532		
Calcium free (apo)					
WT	1.57 ± 2.17	29.31 ± 1.25	1691 ± 1208	-61%	0.006**
C1086R	1.41 ± 1.12	29.02 ± 2.17	1397 ± 896	-80%	< 0.001***
C1111R	2.03 ± 0.75	29.74 ± 1.96	693 ± 275	-66%	0.078
C1117R	2.17 ± 1.88	27.02 ± 2.94	1042 ± 840	-78%	< 0.001***
C1138R	1.36 ± 0.59	29.53 ± 2.07	1027 ± 375	-69%	0.011*

Results averaged from 10 independent 40 ns trajectories recorded following 20 ns of equilibration (mean ± SD). L_o is the distance between the center of mass of cbEGF12 and cbEGF13; L is the average distance. Stiffness multiplied by the average length to calculate apparent modulus. T-test used for mean comparisons.

Table S2. UMD-FBN1 Mutations Database analysis

Disease type	*Custom search (n = 3231)*	*Website search (n = 3077)*	*Difference (%)*
cMFS (n = 1718)			
Classical MFS	1078	1096	18 (1.6%)
MFS	640	640	
nMFS (n = 93)			
Neonatal MFS	79	80	1 (1.3%)
Infantil MFS	14	14	
Excluded (n = 1420)			
NA	789	632	157 (24.8%)
Unknown	102	92	10 (10.9%)
Incomplete MFS	256	255	1 (0.4%)
Probable MFS	20	20	
Classical MFS +	12	12	
Severe MFS	1	1	
Dominant ectopia lentis	111	111	
Isolated skeletal features	10	10	
AAA	14	13	1 (7.7%)
TAAD	26	26	
MASS	5	4	1 (25.0%)
Weill-Marchesani	10	7	3 (42.9%)
Shprintzen-Goldberg	4	4	
Furlong syndrome	1	1	
Lujan-Fryns syndrome	3	3	
Marfanoid syndrome	5	5	
Stiff skin syndrome	5	5	
Ehlers-Danlos syndrome	1	1	
Marfanoid neonatal progeroid syndrome	3	3	
Acromicric dysplasia	13	13	
Geleophysic dysplasia	20	20	
Marfanoid-Progeroid-Lipodystrophy	1	1	
Lens subluxation	8	8	

% difference = | custom search – website search | / website search

Table S3. cbEGF12-cbEGF13 disulfide bond cysteine residue mutations

Domain	*Bond*	*Mutation*	*nMFS*	*cMFS*	*Other*
cbEGF12	1 (C1-C3)	C1074R	2		
		C1074Y	1		
		**C1086R**	**2**		
		C1086Y	3		
		C1086X			1*
	2 (C2-C4)	C1081G	1		
		C1095X			1**
	3 (C5-C6)	**C1111R**	1‡		
		C1111Y			1***
cbEGF13	1 (C1-C3)	**C1117R**	**2**	**1**	
		C1117Y	1	1	
		C1129Y		1	
	2 (C2-C4)	C1124F		1	
		**C1138R**		**2**	
		C1138Y		1	
		C1138G		1	
		C1138S		1	
	3 (C5-C6)	C1140F	1		
		C1140X		1	
		C1153Y			1****
		C1153F		1	
		C1153S	2		

‡C1111R from []; not listed in UMD-FBN1 database; *Dominant ectopia lentis; **NA; ***Incomplete MFS; ****Classical MFS +

Supplementary Video Legends

Video S1. Wild type calcium bound (holo) cbEGF12-cbEGF13 stretched to 50% strain.

Video S2. Wild type calcium free (apo) cbEGF12-cbEGF13 stretched to 50% strain.

Video S3. Wild type calcium bound (holo) TB4-cbEGF23 stretched to +50 Å.

Video S4. Wild type calcium free (apo) TB4-cbEGF23 stretched to +50 Å.

Video S5. C1086R calcium bound (holo) cbEGF12-cbEGF13 stretched to 50% strain.

Video S6. C1086R calcium free (apo) cbEGF12-cbEGF13 stretched to 50% strain.

Video S7. C1117R calcium bound (holo) cbEGF12-cbEGF13 stretched to 50% strain.

Video S8. C1117R calcium free (apo) cbEGF12-cbEGF13 stretched to 50% strain.

Video S9. C1138R calcium bound (holo) cbEGF12-cbEGF13 stretched to 50% strain.

Video S10. C1138R calcium free (apo) cbEGF12-cbEGF13 stretched to 50% strain.

Video S11. C1111R calcium bound (holo) cbEGF12-cbEGF13 stretched to 50% strain.

Video S12. C1111R calcium free (apo) cbEGF12-cbEGF13 stretched to 50% strain.
